# The plant MBF1 protein family: a bridge between stress and transcription

**DOI:** 10.1093/jxb/erz525

**Published:** 2020-02-08

**Authors:** Fabiola Jaimes-Miranda, Ricardo A Chávez Montes

**Affiliations:** 1 CONACyT-Instituto Potosino de Investigación Científica y Tecnológica AC, División de Biología Molecular, San Luis Potosí, San Luis Potosí, México; 2 Unidad de Genómica Avanzada (LANGEBIO), Centro de Investigación y de Estudios Avanzados del Instituto Politécnico Nacional (CINVESTAV-IPN), Irapuato, Guanajuato, México; 3 University of Warwick, UK

**Keywords:** Abiotic stress, heat stress, Multiprotein Bridging Factor 1, stress tolerance

## Abstract

The Multiprotein Bridging Factor 1 (MBF1) proteins are transcription co-factors whose molecular function is to form a bridge between transcription factors and the basal machinery of transcription. MBF1s are present in most archaea and all eukaryotes, and numerous reports show that they are involved in developmental processes and in stress responses. In this review we summarize almost three decades of research on the plant MBF1 family, which has mainly focused on their role in abiotic stress responses, in particular the heat stress response. However, despite the amount of information available, there are still many questions that remain about how plant MBF1 genes, transcripts, and proteins respond to stress, and how they in turn modulate stress response transcriptional pathways.

## Teaser

A review of plant MBF1 proteins, a family of transcriptional co-factors involved in stress responses and stress tolerance.

## Introduction

**Fig. 1.**
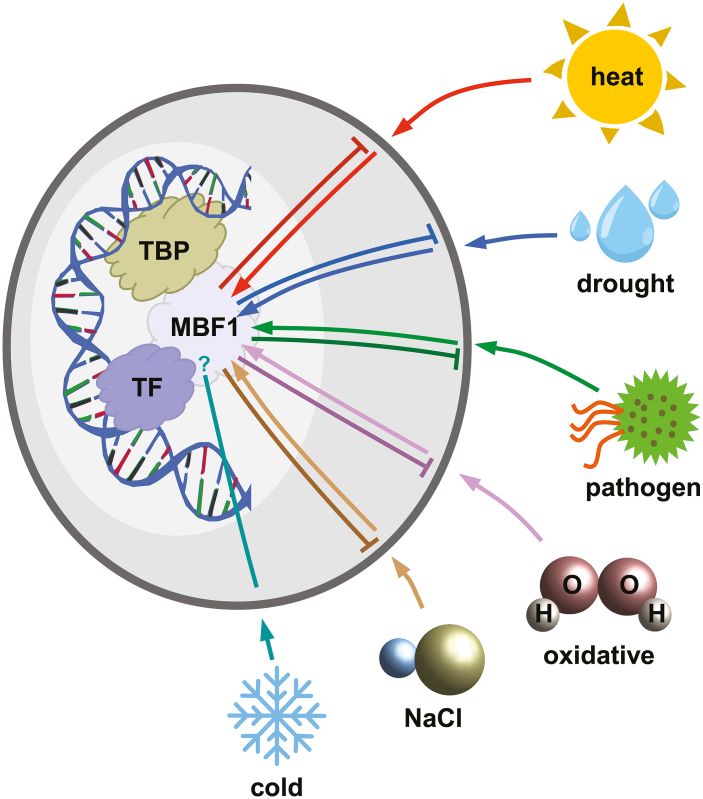
MBF1 proteins and stress responses. MBF1 proteins are transcription co-factors that form a bridge between transcription factors (TF) and the TATA box binding protein (TBP), which is part of the basal transcription machinery. The first transcription factors that were shown to interact with MBF1 belong to the bZIP family, although interactions with other families have been reported. In general, heat, drought, oxidative, salt, and pathogen stress induce up-regulation of MBF1 (depicted as arrows from stress to MBF1), and overexpression of a MBF1 gene usually confers resistance to stress (depicted as blunt arrows from MBF1 to stress). The role of MBF1 in cold stress response is unclear. Some studies strongly suggest that MBF1 proteins could be acting as integrators of multiple and simultaneous stress responses, although the molecular mechanisms through which stresses induce MBF1 up-regulation or MBF1 expression confers resistance to stress are still unknown.

The Multiprotein Bridging Factor 1 (MBF1) family of proteins belongs to the helix–turn–helix superfamily of proteins, which is an ancient superfamily present across the tree of life (Aravind *et al.*, 2005). MBF1 genes are found in most archaea (Aravind and Koonin, 1999; de Koning *et al.*, 2009), and can be found in all eukaryotic genomes available on public databases, either as MBF1 or as endothelial differentiation related factor 1 (EDF1), a common name for animal MBF1 genes. MBF1 was first identified in *Bombyx mori* (silkworm) as a non-DNA-binding transcription co-factor. BmMBF1 forms a bridge between BmFTZ-F1, a transcription factor of the nuclear hormone receptor family and ortholog of the Drosophila regulator fushi tarazu, and the general factor TATA box Binding Protein (TBP), as part of the TATA box protein associated factors (TAF) complex, which is essential for transcription initiation by RNA polymerase II (Li *et al.*, 1994; Fig. 1). Additionally, the MBF1–FTZ-F1 interaction stabilizes FTZ-F1 binding to DNA (Li *et al.*, 1994). Subsequent studies in yeast demonstrated the direct interaction of yeast MBF1 with GCN4, a yeast transcription factor of the basic leucine zipper (bZIP) family, and the formation of a GCN4–MBF1–TBP complex (Takemaru *et al.*, 1998). Takemaru and colleagues determined the relevance of the basic leucine zipper domain for MBF1 binding. They showed that the bZIP domain of GCN4 has homology to the C-terminal domain of the FTZ-F1 DNA-binding region. In particular, they found in this domain four conserved arginines that are required for binding to MBF1 (Takemaru *et al.*, 1998). Other members of the bZIP family, such as bovine Ad4BP/SF1, human ATF1, c-Jun, and c-Fos have been identified as human MBF1 partners (Kabe *et al.*, 1999). Arabidopsis MBF1 proteins are able to bind to yeast GCN4 and yeast TBP in a far-western analysis (Tsuda *et al.*, 2004), which would imply a functional conservation between all eukaryotic MBF1 proteins. Tsuda and colleagues (2004) reported that some members of the Arabidopsis bZIP family retained the arginine at positions considered necessary for MBF1 binding in yeast assays, suggesting that those bZIP proteins could be MBF1 partners in plants. That same year, a protein–protein interaction between a potato (*Solanum tuberosum*) MBF1 and a homeodomain leucine zipper protein (HD-Zip) from sunflower (*Helianthus annuus*), Hahb-4, was described (Zanetti *et al.*, 2004). Hahb-4 is a plant transcription factor that, like other proteins that interact with MBF1, contains a typical leucine zipper motif, and this motif is important for the interaction between Hahb-4 and StMBF1 (Zanetti *et al.*, 2004). This evidence indicates that leucine zipper proteins function as partners of MBF1 across all eukaryotes, and strongly suggests a functional conservation between yeast, animal, and plant MBF1 proteins.

## MBF1 gene and protein structure

### MBF1 gene structure

**Fig. 2.**
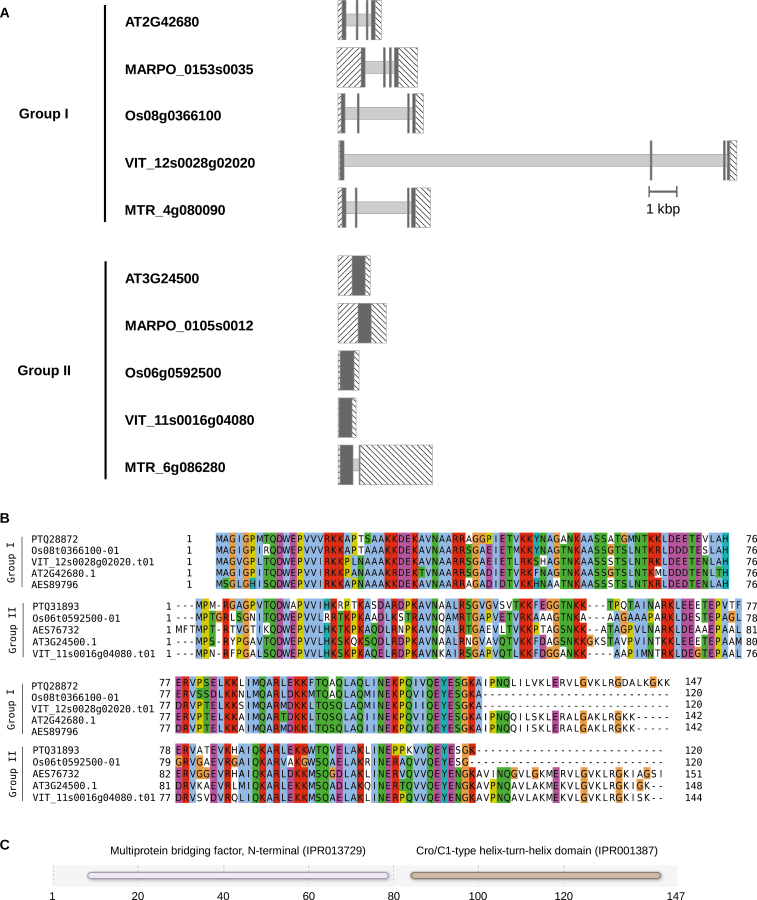
Plant MBF1 gene structure, protein sequence alignment, and protein domain distribution. (A) Examples of group I and group II intron–exon structure for two Arabidopsis (AT2G42680 and AT3G24500), *Marchantia polymorpha* (MARPO_0153s0035 and MARPO_0105s0012), *Oryza sativa* (Os08g0366100 and Os06g0592500), *Vitis vinifera* (VIT_12s0028g02020 and VIT_11s0016g04080) and *Medicago truncatula* (MTR_4g080090 and MTR_6g086280) genes. Hashed boxes represent untranslated region (UTR) sequences, filled boxes represent coding sequences, and lines represent introns. Group I genes contain four exons and three introns. Group II genes do not have this exon–intron structure and can contain none or more introns. UTR and, when present, intron lengths can vary, but coding sequence lengths after splicing are very similar. (B) MBF1 protein sequence alignment. Proteins correspond to the genes in (A). Sequences were downloaded from Ensembl Plants (http://plants.ensembl.org/), aligned with EBI’s MUSCLE (Edgar, 2004) web interface (https://www.ebi.ac.uk/Tools/msa/muscle/), and residues were colored using JalView (Waterhouse *et al.*, 2009). The PTQ28872 protein corresponds to gene MARPO_0153s0035, PTQ31893 to gene MARPO_0105s0012, AES89796 to gene MTR_4g080090, and AES76732 to gene MTR_6g086280. Although group I and group II MBF1 proteins are similar across their primary structure, the alignment shows that both groups are not identical. (C) Domains present in plant MBF1 plant proteins. Plant MBF1 proteins have two domains, an N-terminal domain, appropriately named ‘Multiprotein bridging factor, N-terminal’, and a C-terminal Cro/C1-type helix–turn–helix domain. An Interproscan (Jones *et al.*, 2014 *b*) analysis of the *Marchantia polymorpha* PTQ28872 protein is shown here.

While in non-plant eukaryotes (yeast, fly, mouse, human, etc.) MBF1 is encoded by a single gene, plants have more than one MBF1 gene. For example, the Arabidopsis genome contains three MBF1 genes: *MBF1a* (AT2G42680), *MBF1b* (AT3G58680) and *MBF1c* (AT3G24500), and all three are able to bridge yeast GCN4 and yeast TBP *in vitro*, and to functionally complement an *mbf1* null yeast mutant (Tsuda *et al.*, 2004). Through the construction of a phylogenetic tree using MBF1 protein sequences from 30 species, plant MBF1 proteins were classified into two groups, I and II. Group I is the larger group and includes MBF1a and MBF1b from Arabidopsis. Group II includes MBF1c (Tsuda and Yamazaki, 2004). MBF1 genes that belong to group I possess similar gene structures: four exons and three introns. Group II MBF1 genes do not have this structure, and can contain zero, one, or two introns (Fig. 2A).

### MBF1 protein structure

The primary structure of plant MBF1 proteins is quite conserved, although differences exist between group I and group II sequences (Fig. 2B). MBF1 sequences can be divided in two parts of essentially equal length, which correspond to an N-terminal domain, which has in fact been named ‘Multiprotein bridging factor 1, N-terminal’, and a C-terminal helix–turn–helix domain (Fig. 2C). MBF1 protein function is determined by the secondary structure of both domains, rather than their primary structure. The C-terminal helix–turn–helix domain is the TBP binding domain, and its structure is proposed to be conserved across species. The N-terminal domain is the domain for interaction with transcription factors, and it would appear not to form a defined three-dimensional structure.

#### The MBF1 C-terminal domain is the TBP binding domain

MBF1 protein structure was first studied for the *Bombyx mori* protein. The BmMBF1 C-terminal domain (residues 67–146; MBF1_CTD_) has a defined structure. MBF1_CTD_ is composed of four amphipathic α-helices and a helix–turn–helix motif (Takemaru *et al.*, 1997; Aravind and Koonin, 1999), and was identified as the TBP binding domain (Takemaru *et al.*, 1997; Ozaki *et al.*, 1999). The hydrophobic residues that form the MBF1_CTD_ α-helices are almost completely conserved across MBF1 proteins from *Saccharomyces cerevisiae*, *Bombyx mori*, *Homo sapiens*, and Arabidopsis. This suggests that the structure of MBF1 is essentially identical across species (Ozaki *et al.*, 1999). In a study in yeast, the aspartic acid (D) at position 112 of the third helix was identified as necessary for binding to TBP (Takemaru *et al.*, 1998). ScMBF1–ScTBP interaction is required for transcriptional activation of the HIS3 gene, a histidine auxotrophy selection marker. Yeast D112A MBF1 mutants are sensitive to aminotriazole, an inhibitor of the HIS3 gene product that is used as a diagnostic tool for impaired HIS3 activation (Takemaru *et al.*, 1998). This aspartic acid is conserved between *Bombyx mori*, yeast, human and Drosophila MBF1s. In plants, the aspartic acid is replaced by a glutamic acid, but nevertheless plant MBF1s are able to partially complement a yeast *mbf1* null mutant in the presence of aminotriazole (Tsuda *et al.*, 2004). Liu and colleagues (2007) showed that the MBF1–TBP interaction is conserved across species despite amino acid variations in the TBP binding domain. In addition, in potato, the MBF1_CTD_ domain can be phosphorylated. A phosphorylation region (amino acids 71–117) was identified through *in vitro* and *in vivo* assays, which is phosphorylated by a member of the family of calcium-dependent serine/threonine kinases in response to *Phytophtora infestans* elicitors (Zanetti *et al.*, 2003).

#### The MBF1 N-terminal domain is the transcription factor binding domain

Unlike the carboxyl terminal domain, the N-terminal domain of the human or *Bombyx* MBF1 proteins does not form a single defined structure (Mishima *et al.*, 1999; Ozaki *et al.*, 1999). Ozaki and colleagues (1999) suggested that the structural flexibility of this domain confers to BmMBF1 the ability to adopt different conformations and to interact with different partners. In fact, this region harbors the protein–protein interaction domain that is essential for binding to FTZ-F1 or GCN4 (Takemaru et al., 1997, 1998). In plants, the structure of the N-terminal domain has not been studied. However, i*n vitro* tests demonstrated the direct interaction between each Arabidopsis MBF1 and GCN4 from yeast, which suggests that, even if the N-terminus is not a structured domain, its transcription factor binding function is conserved between animals and plants (Tsuda *et al.*, 2004).

## Plant MBF1 expression patterns and subcellular localization

### Expression patterns

MBF1 expression profiles show a ubiquitous expression of MBF1 transcripts. In Arabidopsis, *MBF1a* (group I), *MBF1b* (group I), and *MBF1c* (group II) expression was found in all tested tissues (leaves, roots, stems, flowers, and siliques; Tsuda *et al.*, 2004). In hot pepper (*Capsicum annuum*), a group I *MBF1* (GenBank JX402927.1) is expressed in root, stem, leaf, flower, fruit, and seed (Guo *et al.*, 2014). In potato, a group I *StMBF1* (GenBank AF232062.1) is expressed in tubers, tuber buds, stems, and fully expanded leaves (Godoy *et al.*, 2001).

While MBF1 transcripts are expressed ubiquitously, differences can be found in expression levels of individual MBF1 transcripts. In Arabidopsis, *MBF1a* is more abundant in flowers, while *MBF1b* is strongly expressed in leaves, stems, flowers, and siliques. *MBF1c* is more abundant in leaves and roots (Tsuda *et al.*, 2004). By histochemical analysis of β-glucuronidase (GUS) activity, it was possible to determine more precisely the expression pattern of each transcript. In 5-week-old Arabidopsis plants, *pMBF1a::GUS* staining was observed in anthers and seeds, *pMBF1b::GUS* in leaf veins, stems, anthers, and seeds, and *pMBF1c::GUS* in leaves, stems, flowers, and siliques (Tsuda and Yamazaki, 2004). A follow-up of MBF1 expression in 3-, 7-, 14-, 21-, and 28-day-old Arabidopsis plants showed that MBF1b is the most expressed MBF1 during development. A strong GUS activity of *pMBF1b::GUS* in leaf veins, petioles, and stems was observed (Tsuda and Yamazaki, 2004), and a more meticulous observation revealed that MBF1b is predominantly expressed in cells around vascular tissues (Tojo *et al.*, 2009). *pMBF1c::GUS* activity was detected in the apical shoot meristem in 14-day-old plants, and in the rest of the tissues in 21- and 28-day-old plants (Tsuda and Yamazaki, 2004). *pMBF1c::GUS* activity was also detected around vascular tissue in leaves, but its expression level was weaker than that of *pMBF1b::GUS* (Tojo *et al.*, 2009). The ubiquitous expression of MBF1 transcripts indicates that these genes are involved in the normal development of the plant. Nevertheless, differential expression of each MBF1 at different plant ages and in the different plant tissues suggests that each MBF1 could be playing different roles at different developmental times.

### Subcellular localization

In *Bombyx mori*, during the larval stage, MBF1 subcellular localization is development dependent. BmMBF1 is present in the cytoplasm, and during one of the intermediate stages of development, called molting D3 stage, it is translocated to the nucleus. After that stage, it is again found in the cytoplasm (Liu *et al.*, 2000). In humans, subcellular localization experiments showed that MBF1 is present in the cytoplasm. However, when MBF1 is co-expressed with the bZIP family protein Ad4BP/SF-1 it migrates to the nucleus (Kabe *et al.*, 1999). The first efforts towards identifying plant MBF1 subcellular location were made in Arabidopsis. Arabidopsis MBF1::GFP constructs for all three proteins were observed in the nucleus, with a strong fluorescence in the nucleolus, and in the cytoplasm (Sugikawa *et al.*, 2005). In cells that were subjected to heat stress, MBF1c::GFP was enriched in the nucleus over the cytoplasm, suggesting that, under heat stress conditions, MBF1c is relocated from the cytoplasm to the nucleus (Suzuki *et al.*, 2008; Kim *et al.*, 2015). This same relocation under heat stress was also observed in wheat (*Triticum aestivum*) cells: a GFP–TaMBF1c fusion protein was largely located in the nucleus, but also in the cytoplasm of onion epidermal cells cultured at 22 °C. However, in cells cultured at 37 °C, GFP–TaMBF1c was mostly present in the nucleus (Qin *et al.*, 2015). Deletion experiments in Arabidopsis and wheat showed that the carboxy terminal domain directs the subcellular localization of the protein (Sugikawa *et al.*, 2005; Qin *et al.*, 2015).

## The biological role of the plant MBF1 family

In plants, MBF1 proteins have been shown to participate in certain developmental processes, but attention has mainly focused on the role of MBF1 in stress responses (Fig. 1; Table 1). Plant MBF1 transcripts are up/down-regulated in response to stress, and plants with altered MBF1 expression present differential responses to stress, usually increased stress tolerance. In the following sections we mention a few examples of MBF1s and development, and then we present what is known about the participation of MBF1 proteins in heat, oxidative, osmotic, drought, cold, and pathogen stress responses.

**Table 1.** Plant MBF1 protein involvement in stress responses and reported protein–protein interactions

Species	Group	Gene or sequence id	Symbol	Involved in stress response								Protein-protein interactions	References
				Heat	Drought	H_2_O_2_	Osmotic	Cold	Drougth+ heat	Osmotic+ heat	Biotic		
Arabidopsis	II	AT3G24500 (TAIR)	MBF1c	Yes	Yes	Yes	Yes		Yes	Yes	*Pseudomonas syringae*	Q9CAR0 (NAC), C0SUZ3 (bZIP), F4I1G5 (non-TF), Q39057 (Zn-finger), Q8LC03 (Homeobox), Q9FRR1 (non-TF)	Rizhsky *et al*. (204); Tsuda *et al.* (2004); Suzuki *et al.* (2005); Kim *et al.* (2007); Arce *et al.* (2010); IntAct^*a*^: Orchard *et al.* (2014); Jones *et al.* (2014a); Trigg *et al.* (2017).
*Dendranthema grandiflorum*	II	unknown	*DgMBF1*			Yes	Yes						Zhao *et al.* (2019)
*Polytrichastrum alpinum*	II	KM978992.1 (GenBank)	PaMBF1c	Yes			Yes						Alavilli *et al.* (2017)
*Pyropia yezoensis*	II	AB480828.1 (GenBank)	PyMBF1	Yes		Yes							Uji *et al.* (2013)
*Retama raetam*	II	AF439278.2 (GenBank)	ERTCA	Yes					Yes				Pnueli *et al.* (2002); Rizhsky *et al.* (2002)
*Triticum aestivum*	II	GQ370008.1 (GenBank)	TaMBF1c	Yes	Yes	Yes							Qin *et al.* (2015)
*Arabidopsis thaliana*	I	AT2G42680 (TAIR)	MBF1a				Yes				*Botrytis cinerea*	C0SUZ3 (bZIP), P93007 (AP2/ERF), Q1PF47 (Zn-finger), Q8RYC8 (ARF), Q9C7E8 (Zn-finger), Q9M274 (NAC)	Kim *et al.* (2007); IntAct^*a*^: Orchard *et al.* (2014); Trigg *et al.* (2017)
*Arabidopsis thaliana*	I	AT3G58680 (TAIR)	MBF1b				Yes					P48002 (Homeobox), Q39057 (Zn-finger), Q3EAI1-2 (bHLH), Q84TK1 (bHLH), Q8L8A5 (SSXT non-TF), Q9FF62 (Zn-finger), Q9M274 (NAC), Q9SRV2-2 (non-TF)	Kim *et al.* (2007); IntAct^*a*^: Orchard *et al.* (2014); Trigg *et al.* (2017)
*Capsicum annum*	I	JX402927.1 (GenBank)	CaMBF1				Yes	Yes					Guo *et al.* (2014)
*Nicotiana tabacum*	I	AB072698.1 (GenBank)	NtMBF1a									Tomato mosaic virus movement protein Q83485	Matsushita *et al.* (2002)
*Solanum tuberosum*	I	AF232062.1 (GenBank)	StMBF1			Yes					*Fusarium eumartii* elicitor	*Helianthus annuus* Hahb-4 (HD-zip); AAA63768.2 (GenBank; Homeobox)	Godoy *et al.* (2001); Arce *et al.* (2006)
*Vitis vinifera*	I	XM_002280956.4 (GenBank)	VvMBF1a		Yes	Yes							Yan *et al.* (2014)

^*a*^ IntAct is a database of protein–protein interactions: https://www.ebi.ac.uk/intact/ (Orchard *et al.* 2014).

### MBF1s and plant development

There are a few reports about MBF1 proteins in the context of plant development. None has specifically addressed their role in this process, although some phenotypes have been described for a few overexpression and mutant lines. Arabidopsis *MBF1c* (group II) overexpression in Arabidopsis resulted in early flowering and an increased number of seeds (Suzuki *et al.*, 2005), and Arabidopsis *MBF1c* overexpression in soybean (*Glycine max*) plants resulted in enhanced yield (Suzuki *et al.*, 2011). In tomato, *ER24* (group II) loss-of-function seeds had an altered germination phenotype (Hommel *et al.*, 2008). Overexpression in Arabidopsis of *MBF1b* or *MBF1c* fused to the strong transcriptional repression domain SRDX resulted in plants with extremely small cotyledons and leaves (Hommel *et al.*, 2008; Tojo *et al.*, 2009). Tojo *et al.* (2009) suggested that MBF1 could negatively regulate endoreduplication, with the reduced ploidy impacting leaf size.

### MBF1s and stress responses

#### MBF1s and heat stress

##### MBF1s participate in heat stress responses

The participation of MBF1 proteins in heat stress responses has been of great interest and has been studied in several plants. Up-regulation of group II MBF1s in plants subjected to heat stress has been observed in Arabidopsis, wheat, the legume *Retama raetam*, the moss *Polytrichastrum alpinum*, and even the red alga *Pyropia yezoensis* (Pnueli *et al.*, 2002; Tsuda and Yamazaki, 2004; Suzuki *et al.*, 2005; Uji *et al.*, 2013; Qin *et al.*, 2015; Alavilli *et al.*, 2017). In Arabidopsis, *MBF1c* messenger accumulation in response to heat stress occurs quite rapidly (Tsuda and Yamazaki, 2004; Suzuki *et al.*, 2008). Accumulation can be observed after 5 min of heat stress (37 °C) and reaches a maximum after 1 h. When a normal growth temperature (22 °C) is restored, *MBF1c* transcript levels also decrease rapidly, and are almost undetectable after 2 h (Tsuda and Yamazaki, 2004). Furthermore, overexpression of a group II wheat MBF1 in rice resulted in plants that were more tolerant to heat stress (Suzuki *et al.*, 2005; Qin *et al.*, 2015). Inversely, Arabidopsis *mbf1c* mutant seedlings were more sensitive to heat stress than are wild-type plants (Suzuki *et al.*, 2008). All these experiments showed that group II MBF1 proteins are associated with heat stress response. The question is whether group II MBF1s are involved in basal or acquired thermotolerance (Bokszczanin *et al.*, 2013). For this, Suzuki and coworkers subjected Arabidopsis wild-type and *mbf1c* null mutant seedlings to either a temperature of 45 °C (basal thermotolerance), or to a two-step heat stress, during which plants were subjected to an intermediate temperature of 38 °C, which allows acclimatization, before being subjected to the more severe temperature of 45 °C (acquired thermotolerance). Null *mbf1c* mutant seedling survival rate was similar to that of wild-type for the two-step heat stress, and expression of acquired response genes, such as those of the HSFA2, HSP70, HSP90, or HSP101 heat shock proteins, was not modified in comparison with wild-type plants, showing that acquired thermotolerance was not compromised in *mbf1c* plants. However, survival of *mbf1c* mutant plants was decreased significantly after a one-step heat stress (Suzuki *et al.*, 2008). Suzuki and colleagues concluded that MBF1c could be part of the basal heat stress response in Arabidopsis, and argued that MBF1c regulates stress response genes, but is not required for the regulation of heat shock proteins (Suzuki et al., 2005, 2008). However, in triple *mbf1abc* knock-down mutant Arabidopsis seedlings, HSP70 was up-regulated, and upon complementation with MBF1c, HSP70 transcript levels returned to wild-type levels, leading to the conclusion that HSP70 is negatively regulated by MBF1c (Arce *et al.*, 2010). Kim and colleagues (2015) have more recently shown that expression of two Arabidopsis heat shock proteins, HSA32 and HSP70T-2, decreased after a two-step heat stress in *mbf1c* mutant Arabidopsis plants.

##### *MBF1 protein–protein interactions during heat stress response*.

In a transcriptome profiling of Arabidopsis plants subjected to heat stress, the relative level of two trehalose-6-phosphate synthases (AT1G70290, TPS8; and AT2G18700, TPS11) was higher in MBF1c overexpression plants compared with wild-type plants (Suzuki *et al.*, 2005). Also, trehalose levels were higher in MBF1c overexpression plants after a heat stress (Suzuki *et al.*, 2005). Trehalose accumulation is involved in enhanced tolerance to abiotic stress (Garg *et al.*, 2002; Penna, 2003), and trehalose is thought to be a signaling molecule in plants during stress (reviewed in Grennan, 2007). Suzuki and coworkers showed that, in Arabidopsis, TPS5 (AT4G17770) is regulated by MBF1c, and that MBF1c regulates trehalose accumulation during heat stress (Suzuki *et al.*, 2008). TPS5 and MBF1c were found to interact in a yeast two-hybrid assay, and null *tps5* and *mbf1c* Arabidopsis single mutants were both sensitive to basal, but not acquired, thermotolerance (Suzuki *et al.*, 2008). TPS5 was localized in the nuclei during heat stress, as was MBF1c, suggesting that both proteins could be interacting *in planta*. These results strongly suggest that there is a link between MBF1c and trehalose metabolism during plant thermotolerance (Suzuki *et al.*, 2008).

Another interesting protein–protein interaction that has been described in Arabidopsis is that between MBF1 and STRESS ASSOCIATED PROTEIN 5 (SAP5, AT3G12630), a member of the stress-associated proteins. SAP5 is located in the nucleus and has been associated with heat stress responses (Kim *et al.*, 2015). When it was overexpressed, MBF1c transcript levels increased (Kim *et al.*, 2015). Bimolecular fluorescence complementation assays in Arabidopsis mesophyll protoplasts and tobacco epidermal leaf cells showed that SAP5 and MBF1c interact in the nucleus. In *in vitro* experiments using the luciferase reporter fused to HSP18.2 promoter as a stress-inducible molecular chaperone involved in basal and acquired heat tolerance in Arabidopsis, luciferase activity was significantly more induced when MBF1c and SAP5 were co-expressed during a heat treatment than when only SAP5 or MBF1 was present, or when neither was present (Kim *et al.*, 2015).

#### MBF1s and oxidative stress

MBF1s are also involved in oxidative stress response. H_2_O_2_ treatments lead to an increase in group II transcript up-regulation in *Pyropia yezoensis* cells (Uji *et al.*, 2013), Arabidospsis seedlings (MBF1c; Tsuda and Yamazaki, 2004), and wheat seedlings (Qin *et al.*, 2015). A potato group I MBF1 is also induced after a H_2_O_2_ treatment of cell suspensions from friable calli (Arce *et al.*, 2006). When a grape (*Vitis vinifera*) group I MBF1 was overexpressed in Arabidopsis, the plants accumulated less reactive oxygen species (O_2_^−^ and H_2_O_2_) than wild-type plants after a drought stress, and suffered less oxidative damage (Yan *et al.*, 2014). Overexpression of a group II MBF1 from chrysanthemum (*Dendranthema grandiflorum*), *DgMBF1*, resulted in plants that accumulated less H_2_O_2_ and O_2_ during a NaCl treatment, and increased antioxidant enzyme activity compared with wild-type plants (Zhao *et al*., 219).

Conversely, in Arabidopsis *mbf1abc* triple mutant plants, the ability to contend against an oxidative stress is severely impaired. Roots and cotyledons of this triple mutant enter cell death after a treatment with H_2_O_2_, and germinating seeds were strongly sensitive to methyl viologen, an inductor of oxidative injury through O_2_^−^ production (Arce *et al.*, 2010). These phenotypes were partially reverted by *MBF1c* overexpression (Arce *et al.*, 2010). Additionally, Arce and colleagues (2010) found that, in Arabidopsis, MBF1s are associated with regulation of expression of the *ABA REPRESSOR1* (*ABR1*; AT5G64750) gene under normal growth conditions and MV-induced stress conditions. *ABR1* is strongly induced by several stress conditions, and *abr1* mutants are hypersensitive to ABA, osmotic stress, and glucose (Pandey *et al.*, 2005). These results suggest that MBF1s could participate in the prevention of the damage caused by oxidative stress.

#### *MBF1s and osmotic*, *ionic and salinity stress*

MBF1s have also been associated with salt, ionic, and osmotic stresses. During the first studies on the role of MBF1 in the stress response in Arabidopsis, Tsuda and Yamazaki (2004) observed that MBF1c was up-regulated in seedlings after a NaCl treatment. Later, in a larger study in Arabidopsis, Kim and colleagues (2007) observed that all three MBF1s increased their expression in response to a NaCl treatment in 2-week-old plants. However, MBF1a (group I) was the most expressed transcript, and its up-regulation occurred earlier than that of MBF1b or MBF1c (Kim *et al.*, 2007). In chrysanthemum, *DgMBF1* transcript levels increased after a NaCl treatment, and overexpression of *DgMBF1* in chrysanthemum resulted in enhanced tolerance to salt stress (Zhao *et al*., 219). Also, overexpression of MBF1a in Arabidopsis increases seedling tolerance to salt stress, and induces the expression of *RD29A* (AT5G52310), *RD22* (AT5G25610), and *KIN2* (AT5G15970). These genes are involved in dehydration, cold (*RD29A*), and dehydration, cold and ABA responses (*RD22*, *KIN2*) (Kim *et al.*, 2007). Additionally, MBF1c overexpression in Arabidopsis confers tolerance to osmotic stress in seedlings (Suzuki *et al.*, 2005). During germination at high sorbitol concentrations, triple mutant *mbf1abc* seeds germinated 60% less than wild-type seeds, and this phenotype was completely rescued by overexpression of MBF1c. These results reinforce the idea that MBF1c participates in the osmotic stress response and confers tolerance to this stress during germination (Arce *et al.*, 2010).

In the antarctic moss *Polytrichastrum alpinum*, *PaMBF1c* (group II) transcript levels increased after NaCl and mannitol treatments, and *PaMBF1c* overexpression in Arabidopsis increased plant tolerance to mannitol, salt (NaCl), and ionic (LiCl) stress (Alavilli *et al.*, 2017). Furthermore, a comparison of the overexpression in Arabidopsis of either MBF1c of *P. alpinum* or MBF1c of Arabidopsis resulted in interesting observations. *PaMBF1c* overexpression in Arabidopsis was able to increase osmotic, ionic, and salt stress tolerance during germination more than was *MBF1c* overexpression, and *PaMBF1c* overexpression seedlings were more tolerant to salt and ionic stress than *MBF1c* overexpression seedlings. It is interesting to note that *P. alpinum* lives under severe stress conditions, and that PaMBF1c overexpression in Arabidopsis confers more tolerance to different stresses than Arabidopsis’s own MBF1c overexpression. An RNA-seq comparison of both overexpression lines under salt stress conditions showed that 14 genes were exclusively up-regulated in PaMBF1c overexpression plants. Among these were genes involved in the production and transport of ATP as well as the ATP-dependent Ca^2+^ pump. ATP has been related to oxidative stress and this to salt stress. Functions such as ROS detoxification, maintenance of ATP homeostasis, and facilitation of Ca^2+^ signaling could be enhanced in PaMBF1c overexpression plants, rendering them more tolerant to salt stress (Alavilli *et al.*, 2017).

There are also examples in which MBF1 proteins do not appear to participate in osmotic or ionic stresses, or can even lower the tolerance to such stresses. In wheat, *TaMBF1c* transcript levels were not affected by a NaCl treatment (Qin *et al.*, 2015). In hot pepper, contrary to what has been observed in other plants, *CaMBF1* transcript levels dramatically decrease in response to several stimuli, such as salicylic acid, NaCl stress or osmotic stress. Furthermore, *CaMBF1* overexpression in Arabidopsis affects plant responses to high salt concentrations: germination is delayed, and seedlings are hypersensitive to NaCl (Guo *et al.*, 2014).

#### MBF1s and drought stress

Arabidopsis, wheat, and rice group II, and *Vitis vinifera* group I *MBF1* transcript levels increase in response to drought (Rizhsky *et al.*, 2004; Tsuda and Yamazaki, 2004; Suzuki *et al.*, 2005; Ray *et al.*, 2011; Baloglu *et al.*, 2014; Yan *et al.*, 2014; Qin *et al.*, 2015). *VvMBF1* expression was significantly induced after a drought treatment. When plants were re-watered, *VvMBF1* expression declined until it reached levels equal to those of untreated controls (Yan *et al.*, 2014). Water loss in plants is regulated by stomatal aperture, which is in turn regulated by the hormone ABA. Overexpression of *VvMBF1* in Arabidopsis led to stomatal closure, suggesting that MBF1c could be regulating water loss via ABA (Yan *et al.*, 2014). Furthermore, Arabidopsis MBF1c (group II) and grape MBF1 (group I) transcript levels increased in response to ABA (Tsuda and Yamazaki, 2004; Yan *et al.*, 2014), and VvMBF1 overexpression in Arabidopsis resulted in up-regulation of ABA-dependent (RD22 and RD29B), but not of ABA-independent (RD29A and ERD1) drought stress response genes (Yan *et al.*, 2014).

#### MBF1s and cold stress response

The role of MBF1s in the cold stress response has not been clearly defined. In Arabidopsis seedlings, cold stress does not affect MBF1c expression levels, and MBF1c overexpression does not seem to have any effect on seedling tolerance to cold stress (Suzuki *et al.*, 2005). The expression of *R. raetam*’s ERTCA (group II; see next section) is also not affected by cold stress (Pnueli *et al.*, 2002). However, overexpression of a group I hot pepper MBF1 in Arabidopsis increased sensitivity to cold stress. Furthermore, some genes induced in cold stress response, such as those for RD29A, ERD15 and KIN1, were down-regulated in *CaMBF1* overexpression plants (Guo *et al.*, 2014). It would appear that MBF1 works as a negative regulator of cold stress response in hot pepper (Guo *et al.*, 2014).

#### MBF1s can integrate responses from different abiotic stresses

In *R. raetam*, a desert evergreen legume common to arid ecosystems around the Mediterranean basin, a group II MBF1 gene named *ethylene-response transcriptional co-activator* (*ERTCA*) was identified as responding to climatic conditions. *ERTCA* is normally induced in non-dormant plants in the hottest and driest months, and particularly during the hottest hours, when temperatures of 40 °C can be reached (Pnueli *et al.*, 2002). These observations suggest that MBF1 acts as a regulator of the combination of heat and drought stresses, a common stress combination for plants that grow in extreme conditions of heat and low water availability (Rizhsky *et al.*, 2002; Suzuki *et al.*, 2005). In tobacco and Arabidopsis, the expression of group II MBF1 transcripts is higher in plants that are submitted to a combination of heat and drought stress than in plants submitted to a single heat or drought stress. Also, in Arabidopsis, MBF1c overexpression seedlings were more tolerant than wild-type plants to heat stress, osmotic stress and a combination of heat and osmotic stress (Suzuki *et al.*, 2005). However, MBF1 overexpression does not always confer increased tolerance to a combination of stresses. For example, while MBF1c overexpression seedlings are more tolerant to salt stress than wild-type plants, salt stress tolerance is not maintained when MBF1c overexpression seedlings are subjected to a simultaneous salt and heat stress (Suzuki *et al.*, 2005). Furthermore, *mbf1c* mutant plants are not more sensitive to a combined heat and salt stress than wild-type plants (Suzuki *et al.*, 2016). Thus, group II MBF1s appear to be associated to plant tolerance to a combination of heat and drought stress, but not to a combination of heat and salt stress (Suzuki *et al.*, 2016).

### MBF1s and plant pathogen response

There are several pieces of evidence that suggest that MBF1 proteins are involved in response to pathogen attack. When cell suspension cultures of *Solanum tuberosum* L. cv. Spunta were exposed to an elicitor from the fungus *Phytophthora infestans*, StMBF1 (group I) was phosphorylated (Zanetti *et al.*, 2003). StMBF1 was also up-regulated in potato tubers during an infection by *Fusarium eumartii*, and after treatment with a *Fusarium eumartii* elicitor. Also, *StMBF1* expression in potato tubers was increased in response to wounding, ethephon (an ethylene precursor) and salicylic acid, but not to methyl jasmonate, all of which are associated with pathogen response (Dong, 1998). These results link StMBF1 and the response against fungal infection via ethylene and salicylic acid (Godoy *et al.*, 2001).

Further evidence of MBF1 protein involvement in pathogen response was found in Arabidopsis. Suzuki and colleagues (2005) found that proliferation of *Pseudomonas syringae* was lower in Arabidopsis MBF1c overexpression plants than in wild-type plants 48 h after inoculation. Additionally, MBF1a (group I) transcript levels increased in response to a *Botrytis cinerea* infection, and MBF1a overexpression plants had minor lesions compared with wild-type plants during an infection (Kim *et al.*, 2007). Expression of some genes that have been identified as pathogen defense genes, such as PDF1.2, which is regulated by ethylene and jasmonic acid, and PR2, which is regulated by salicylic acid, were up-regulated in MBF1a overexpression plants (Kim *et al*., 207). These results suggest that MBF1a could be providing protection to the plant against pathogen attack via ethylene and jasmonic acid-dependent pathways (Kim *et al.*, 2007). Finally, tobacco (*Nicotiana tabacum*) group I NtMBF1a was identified as a protein–protein interactor of the tomato mosaic virus movement protein. This opens the possibility that these proteins can modulate the response to plant infection by binding to MBF1, suggesting that MBF1 proteins are also key defense proteins (Matsushita *et al.*, 2002).

## Possible avenues for future plant MBF1 research

The participation of plant MBF1s in stress responses, in particular the heat stress response, has been well documented. However, we are yet to identify the molecular mechanisms through which MBF1 proteins respond to stress and in turn modulate stress responses. MBF1s are transcription co-factors, and one can easily imagine how the flexibility of the N-terminal domain allows an MBF1 protein to bind to different partners as the cellular environment changes, for example from a non-stress condition to a heat stress condition, leading to changes in gene the expression profiles. Furthermore, acting as a transcription co-factor might not be the only molecular function of MBF1 proteins. The yeast MBF1 protein prevents frameshifting of stalled ribosomes (Hendrick *et al.*, 2001, Wang *et al*., 2018) and is able to bind RNA (Klass *et al.*, 2013). Are plant MBF1 proteins also able to prevent frameshifting or bind RNA? Could they also bind DNA? In an intriguing piece of work, Suzuki and colleagues (2011) propose that Arabidopsis group II MBF1c could act as a bona fide transcription factor. MBF1c contains a putative helix–turn–helix DNA-binding domain, and systematic evolution of ligands by exponential enrichment, gel mobility shift assays and transcriptional activation in yeast showed that it can bind to specific DNA sequences. So far, we do not know if group I MBF1 proteins are also able to bind DNA. Yet, a transcription factor molecular function for MBF1 proteins would imply that they can regulate the transcription of both the target genes of their transcription factor protein–protein partners and their own. Are the protein–protein partners of different MBF1 proteins from the same organism also different? Also, if all MBF1 proteins are indeed transcription factors, do they bind distinct DNA motifs? The answer to the latter question would probably be yes, as Arabidopsis group I and group II MBF1 proteins are only 55% identical across the proposed DNA-binding domain (Suzuki *et al.*, 2011).

Plant genomes contain more than one MBF1 gene, and their expression, while ubiquitous, is differential. Additionally, there can be different splicing versions for a single MBF1 gene. In fact, even in humans for a single MBF1 gene there are two differentially expressed splice variants (Kabe *et al.*, 1999). Differential expression adds a second layer of versatility to MBF1 proteins. How is plant MBF1 gene expression regulated? Are splice variants also differentially expressed? Numerous pieces of evidence show that stress conditions can modulate MBF1 gene expression. What are the transcriptional pathways triggered from stress perception to MBF1 gene expression to MBF1-regulated stress responses?

MBF1 proteins have been shown to be involved in abiotic and biotic single stress response pathways, but also in the response to simultaneous stresses, as is the case for *R. retam*’s ERTCA and *P. alpinum*’s PaMBF1c. Stresses in nature are more likely to occur simultaneously rather than as punctual, isolated incidents. The molecular versatility of MBF1 proteins therefore makes them ideal candidates to be the integrators of multiple stress conditions. How do MBF1 proteins integrate simultaneous stress stimuli? And how do MBF1 proteins coordinate stress responses with developmental pathways? All these are the next questions to be answered in our journey towards understanding the biological role of the plant MBF1 protein family.

## Abbreviations

bZIP: basic leucine zipper
MBF1: Multiprotein Bridging Factor 1
TBP: TATA box binding protein
